# Complete Genome Sequence of Rhizobium sp. Strain 11515TR, Isolated from Tomato Rhizosphere in the Philippines

**DOI:** 10.1128/MRA.00903-18

**Published:** 2018-08-23

**Authors:** Andrew D. Montecillo, Asuncion K. Raymundo, Irene A. Papa, Genevieve Mae B. Aquino, Albert Remus R. Rosana

**Affiliations:** a Microbiology Division, Institute of Biological Sciences, University of the Philippines Los Baños College, Laguna, Philippines; b Departament de Produccio Vegetal I Ciencia Forestal, Universitat de Lleida, Lleida, Spain; c National Academy of Science and Technology Philippines, Taguig City, Philippines; d National Institute of Molecular Biology and Biotechnology, University of the Philippines Los Baños College, Laguna, Philippines; e Office of the Vice Chancellor for Research and Extension, University of the Philippines Los Baños, College, Laguna, Philippines; f Department of Chemistry, University of Alberta, Edmonton, Alberta, Canada; University of Maryland School of Medicine

## Abstract

Rhizobium sp. strain 11515TR was isolated from the rhizosphere of tomato in Laguna, Philippines. The 7.07-Mb complete genome comprises three replicons, one chromosome, and two plasmids, with a G+C content of 59.4% and 6,720 protein-coding genes. The genome encodes gene clusters supporting rhizosphere processes, plant symbiosis, and secondary bioactive metabolites.

## Abstract (precis)

Rhizobium sp. strain 11515TR was isolated from the rhizosphere of tomato in Laguna, Philippines.

## ANNOUNCEMENT

Rhizobia are best known as nitrogen-fixing bacteria that may form root nodules on leguminous plants. They belong to the superfamily Rhizobiaceae in the alphaproteobacterial superphylum consisting of phenotypically diverse member genera such as Bradyrhizobium, Ensifer (formerly Sinorhizobium), Mesorhizobium, and Rhizobium (1). The mosaic structure of their symbiotic genome compartments could give rise to several rhizobial species colonizing diverse plant hosts, which may have arisen through lateral gene transfer of symbiotic genes (2, 3).

Here, we report the complete genome of Rhizobium sp. strain 11515TR, a novel isolate from tomato rhizosphere in Los Baños, Laguna, Philippines. The strain was grown in yeast mannitol medium (Sigma-Aldrich, Germany) at 25°C for 24 to 48 h. Genomic DNA was extracted using an MGmed (Republic of Korea) DNA purification kit according to the manufacturer’s protocol. The whole genome was sequenced by Macrogen, Inc. (Republic of Korea) using a PacBio RS II platform, which generated 334,388 reads (*N*_50_ = 7,905 bp) from a 20-kb library. The reads were *de novo* assembled using the Hierarchical Genome Assembly Process version 3.0 (HGAP3; Pacific Biosciences) (4), which generated three closed contigs (*N*_50_ = 4,003,789 bp) estimated at 7,070,317 bp. The multipartite genome revealed that the strain has three replicons, one chromosome and two plasmids (p11515TR-A and p11515TR-B), with sequencing coverages of 141×, 134×, and 145×, respectively. Genome annotation was performed independently using the NCBI Prokaryotic Genome Annotation Pipeline (PGAP) (5) and the Joint Genome Institute–Integrated Microbial Genomes and Microbiomes (JGI-IMG/M) pipeline (6). Genome annotation of the combined replicons identified 6,720 protein-coding genes, 67 RNAs, and a G+C content of 59.4%. The sequences were classified into 493 subsystems covering 44% of the genome. The species was established by using the Microbial Genome Atlas (MiGA) (7) in comparison to the NCBI RefSeq and prokaryotic databases, calculating the average nucleotide identity (ANI) using the ANI calculator (8), and determining the digital DNA-DNA hybridization (dDDH) using the Genome-to-Genome Distance Calculator version 2.1 (9). Secondary bioactive metabolites were predicted using antiSMASH version 4.0 (10).

The MiGA identified the type strain R. tropici CIAT 899 (ANI = 90.2%, dDDH = 3.3%) and R. lusitanum P1-7 (ANI = 89.7%, dDDH = 0.8%) as the closest genomes to that of strain 11515TR. The calculated values are below the species cutoff (ANI = 95%, dDDH = 70%) (6, 8), supporting the systematic placement of strain 11515TR in the genus Rhizobium but suggesting a novel Rhizobium species. The genome revealed gene inventories supporting rhizosphere processes, including the presence of nodulation genes (*nod* and *nol*) (11), nitrogen fixation genes in p11515TR-B, chemotaxis genes in p11515TR-A, exopolysaccharide biosynthesis, and quorum-sensing molecules such as homoserine lactone. The genome also encodes gene clusters potentially involved in symbiotic association with plants, such as auxin biosynthesis, lignin degradation, and both type IV and VI secretion systems. Several toxin-antitoxin systems putatively involved in programmed cell death were further detected. Finally, several gene clusters for secondary bioactive metabolites were also predicted from the genome, including vicibactin, terpenoid, polyketide synthases, and nonribosomal peptide synthetase.

### Data availability.

The whole-genome shotgun project reported here has been deposited at DDBJ/EMBL/GenBank under the accession numbers CP022998, CP022999, and CP023000 for the chromosome, plasmid p11515TR-A, and plasmid p11515TR-B, respectively. The versions described in this paper are the first versions.
